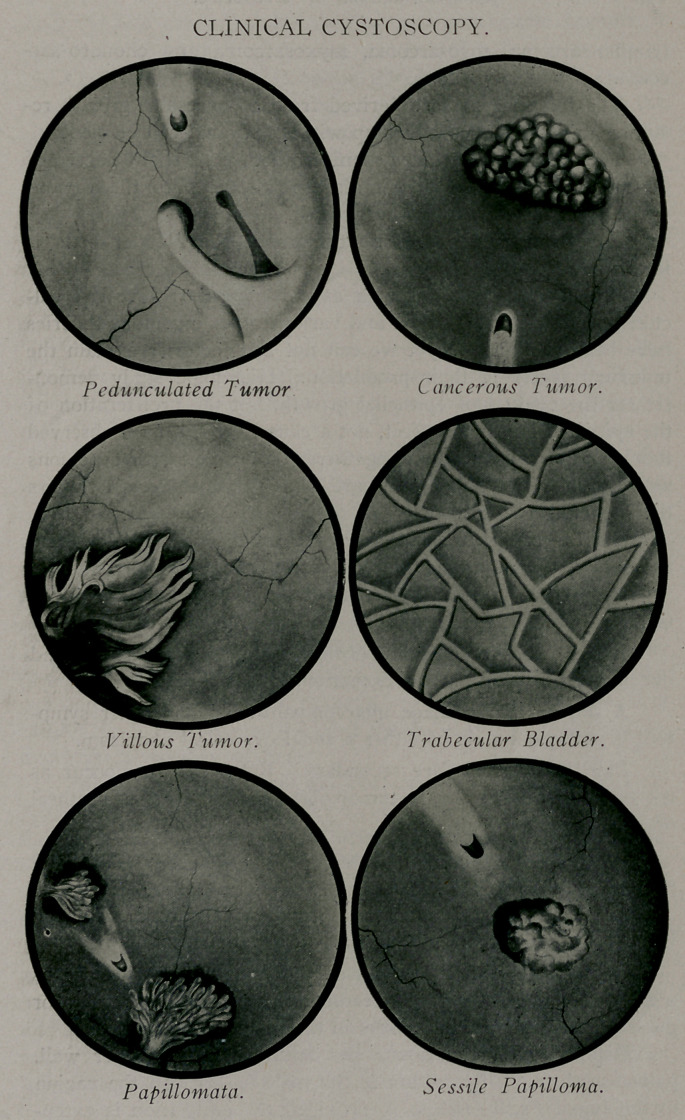# Tumors of the Bladder

**Published:** 1908-06

**Authors:** Alfred L. Fowler

**Affiliations:** Atlanta, Ga.; Clinical and Didactic Lecturer on Genito-Urinary Surgery and Venereal Diseases in the Atlanta College of Physicians and Surgeons; Physician and Surgeon to the United States Penitentiary Hospital; Surgeon to St. Joseph’s Infirmary; 928-929 Candler Building


### TUMORS OF THE BLADDER.*

BY ALFRED L. FOWLER, M. D., ATLANTA, GA.

Clinical and Didactic Lecturer on Genito-Urinary Surgery and Venereal Diseases in the Atlanta College of Physicians and Surgeons; Physician and Surgeon to the United States Penitentiary Hospital; Surgeon to St. Joseph's Infirmary.

The word tumor is a very ancient name and simply means a swelling. Clinically, the word is not likely to disappear, notwithstanding it has lost its importance to the pathologist.

Our knowledge of tumors of the bladder, the result of long continued pathological and experimental research, is a modern acquisition and the perfection of our diagnostic means is even more recent.

A satisfactory classification of these growths, in every respect, is difficult. The most natural method is to divide them into two classes, benign and malignant But even this is attended with great difficulties.

Of those of the first class *papilloma* is the more common and which may be sessile or pedunculated, single or multiple. This class is generally multiple and grows from a common pedicle as do the branches of a tree. These branches, or villi, are composed of a fine stroma of connective tissue, each branch having a loop of blood vessels extending to its extremity, and covered by several layers of epithelium.

When the bladder is distended with fluid these processes unfold and float like aquatic plants in deep water, as they are seen to do when viewed through the cystoscope.

When the connective tissue stroma is particularly well developen it is called a *fibrous papilloma* and is typical because a collection of papillary formations rests upon a more or less thick pedicle; and when the branches are composed of thread-like processes, so gracefully described by Thompson, it is known as a *fimbriated papilloma.*

Clinically, these tumors are generally benign or, as the French aptly say, good natured. But we must bear in mind that this is

*Read before the Georgia Medical Association, Fitzgerald, April 15-16-17, 1908.

not always so. They may be benign in their superficial portion while their deeper parts or base is malignant.

In the benign or good natured growths the connective tissue stroma arises directly from the mucosa or sub-mucosa, while in villous carcinoma there is a small-celled infiltration into its base, and an irregular proliferation of epithelium penetrating its deeper parts.

Villous papillomata are met with anywhere in the bladder, even in a diverticulum, but generally they are situated about the trigonum.

Among the growths of the second class and of greatest importance are, *Carcinoma* and *Sarcoma,* which are met with frequently. They occur in various forms and are seen as huge masses growing into the vesical cavity or even so minute as a slight infiltration of the bladder wall.

Of the two, carcinoma is the more common. It may arise in the form of hard or medullary nodules or it may appear as a diffuse flat infiltration, involving an extensive area of the bladder wall.

Villous carcinoma may occur primarily in the renal pelvis and secondarily in the bladder as a result of villous tufts becoming detached and transported to the bladder where they become implanted.

We distinguish, according to their structure, the scirrhus, medullary, and alveola types, cancroid and melanoma. The epithelial layers, histologically, are usually the starting point, less frequently the glands of the mucosa.

Of these the carcinoma simplex, usually termed scirrhus, is the most frequent form met with, and in which there is an abundance of fibrous tissue elements, while the specific cell elements are less prominent. Those rich in cell elements, carcinoma medullare, represent the softer forms, and are by no means rare. The most obvious characteristic of this growth is its great tendency towards ulceration at an early date.

The alveolar, or gelatinous cancer, particularly malignant, shows a characteristic colloid degeneration of the epithelium.

The cancroid contains the typical epithelial pearls.

The pigment cancer, melanoma, is characterized by a deposit of black pigment in its cells.

Sarcoma is one of the rarest growths of the bladder. Out of eighty-two vesical tumors Albarran only found two cases of sarcoma.

This growth is usually seated in the fundus and is composed of spindle or round cells. Borst divides this form of growth into simple sarcoma and the highly developed sarcoma. In the former, he includes the round cell and spindle cell sarcoma, while in the more highly developed types he includes fibro-sarcoma,

---

### TUMOR OF THE BLADDER.

*Case Mr. A. W. H. (Death from Hemorrhage) Showing Sessile Papilloma Near Left Ureter.*

### CLINICAL CYSTOSCOPY.

*Pedunculated Tumor*

*Cancerous Tumor.*

*Villous Tumor.*

*Trabecular Bladder.*

*Papillomata.*

*Sessile Papilloma.*

lympho-sarcoma, myosarcoma, myxosarcoma and chondro-sarcoma.

The former types are derived from an excessive growth resulting in a secondary filling up with embryonal cell elements.

The second group show manifestations of a slight degree of degeneration, unmistakable in their relationship to the various tissue types of normal tissue growth.

Concerning the causes of tumors of the bladder little is known.

Prolonged irritations, such as chronic catarrh, vesical calculus, prolonged catheterism and various other ingenious theories have been propounded yet we can not satisfactorily explain the unknown factor in their production. Histologic study demonstrates the method of epithelial growth, but the proliferation of the epithelial bud is an effect, not a cause. It has been observed too long, perhaps, to be altogether a coincidence, that persons working in dye establishments are more prone to vesical tumors than others.

*Symptoms.*—In the majority of cases the first, the last, and the only symptom of a tumor of the bladder is hemorrhage.

It may be stated, clinically, that the more villous the tumor the more profuse the bleeding.

In tumors covered with a normal membrane, as myoma and fibroma, hemorrhage is the exception.

A profuse hemorrhage unaccompanied by any other symptom is pathognomonic of either a renal or vesical neoplasm.

Pain and dysuria are secondary. Retention may occur as the result of a large clot or tumor obstructing the vesico-urethral opening.

Cystitis too, is generally secondary to the hemorrhage and dysuria is concomitant to it.

The urine may be entirely normal or it may contain red blood cells, macroscopically or microscopically.

Occasionally small particles of the tumor, even as large as a pea, are voided. This happens now and then in papillomata or good natured tumors, but rarely in the malignant growths.

*Diagnosis.*—If the tumor has infiltrated the bladder wall, palpation through the rectum in the male or through the vagina in the female, will disclose it, provided the infiltration is extensive. If not extensive this method proves negative because it may be so superficial that it can not be felt.

On the other hand cystoscopy gives us positive and reliable results. It is in diagnosticating tumors of the bladder that the cystoscope has proved so triumphant. Usually it requires only a glance to determine their presence. Further, we actually see the tumor, its size, shape, and location and whether it is villous, pedunculated or sessile.

It is an object of much attention for us to bear in mind that unless the exact location of every villous tumor is definitely determined by cystoscopy, before operating, some of them will be overlooked.

We know that many of them may be seen floating in the liquid medium yet when the bladder is opened unless their location is known beforehand, they are frequently undiscoverable.

For determining the site of villous growths, which by the way are seen as beautiful fantastic creations, apparently growing upon a sandy shore, the irrigating cystoscope should be employed because its stream causes a continuous change in the field of vision thereby facilitating our study of the condition and location of the tumor's pedicle, and which is of great value to us subsequent to operation.

While it is generally true that we nearly always find vesical tumors situated adjacent to either the mouth of the urethra or one of the ureter openings, nevertheless it is advisable for us to employ both the direct view and the prismatic cystosocopes to avoid the possibility of error.

For examining the base and posterior wall of the bladder an instrument of the Cabot type is to be preferred. For examining the fundus the Bierhoff-Frank irrigation Cystoscope or the Otis observation cystoscope is more desirable, while if the anterior wall and prostate are to be examined the retragrade cystoscope will serve our purpose best.

The characteristics of many of the different vesical tumors, as seen through the cystoscope, have been very beautifully reproduced for me from cystoscopic photographs by Mr. S J. Bernolak who has taken sufficient interest in this line of work to examine with me some of the pathologic changes occurring in the bladder. The interior of the bladder and its contents are easily photographed by attaching a miniature camera to the ocular end of the cystoscope. Perhaps the Nitze camera manufactured by L and H. Lowrenstein, Berlin, is the most serviceable as it is detachable and can be applied to any cystoscope. The Crammer Dry Plate Company of St. Louis, manufacture plates for it which only require 5 or 10 seconds exposure in a well illuminated bladder.

The different types of cystoscopes mentioned I take pleasure in handing you for your inspection.

If the tumor be in one of the ureters or kidney and not in the bladder, and blood be coming from it, the cystoscope will reveal the side involved because the blood can be seen issuing from the uteral opening of that side. Moreover, the ureters are easily catheterized by those of us who have given such work the requisite time and patience, and in this way only the urine from either kidney may be studied separately.

Cystoscopy, when performed in a diseased bladder; also obtaining the separate urines by ureteral catheterization, calls loudly for a professional cystoscopist and in the hands of a neophyte the cystoscope is an instrument to be dreaded. One case is reported simply to accentuate its dangers when in the hands of an inexperienced person.

*Sessile Papilloma.*—Mr. A. W. H., age 58; married; occupation, insurance clerk. About eight months ago patient discovered that he was passing clots of blood in his urine. His family physician, whom he consulted at that time, expressed the opinion that they were coming from the urethra. An internal medication was prescribed by his physician and the blood and clots ceased about as suddenly as they had appeared. Urine remained clear until August third, when blood and clots were passed three or four times daily for three days. Patient's physician ordered him to bed and prescribed adrenalin chloride internally, and irrigated his bladder daily with hot boric acid solution. Hemorrhage completely disappeared August 9th for a day, only to return in greater quantity the following day. His attending physician at first suspected the prostate as the probable cause of the hemorrhage, but as the prostate was about normal in size he was at a loss how to account for the hemorrhage in this connection. In his efforts to diagnosticate the cause he attempted to use an air dilating cystoscope, with which he was unfamiliar and with which he stated he was unable to make out anything. Unskilful cystoscopy aggravated the hemorrhage and it had become serious. It was at this juncture that I was called into consultation. From all the data the physician was able to supply, I reasoned by exclusion, that in all probability the patient had a villous tumor. The next day his physician returned to my office stating that the patient's vitality was beginning to fail and that he was fearful of losing him. I recommended an aluminum sulphate irrigation, stating that if it failed to do good, a suprapubic cystotomy was the patient's only chance. To this the physician stated that he did not think the patient was in condition to stand a general anesthetic. I suggested local anesthesia, which had not occurred to the doctor, he remarking that that threw a new light upon the situation. Over two days were lost before family consented to the operation; meanwhile the patient was still passing clots and blood. We preformed a suprapubic cystotomy under local anesthesia. The bladder was swollen, protruded considerably above the pubic bone and was suggestive of the gravid uterus. Over two pints of clotted blood were removed. Bladder was irrigated with a hot saline solution and packed with gauze, wrung out in adrenalin chloride solution. Up to this time the patient had stood the operation well, but a quarter of an hour later a "sledge-hammer pulse" came on and the patient became irritable. No noticeable change for the next two hours, when the patient steadily declined and died—four hours after the operation. By the aid of a small incandescent lamp introduced through the suprapubic incision the autopsy disclosed a sessile papilloma nearly the size of a dime, located about an inch to the left of the left ureter, and which was not observed in the hurry of the operation.

To my mind this case illustrates very forcibly three important points: The danger of delay in desperate hemorrhage. The danger in clumsy cystoscopy. The danger of over stimulation from adrenalin chloride, particularly in a bladder that has lost its tone.

The treatment of tumors of the bladder is purely surgical and perferably by the supra-pubic route. Palliative measures are a waste of time and surgical intervention, in the vast majority of cases, is the patient's onlv hope.

928-929 Candler Building.

Bibliography: *Keys, Lowenheim and Bland-Sutton.*

## Figures and Tables

**Figure f1:**
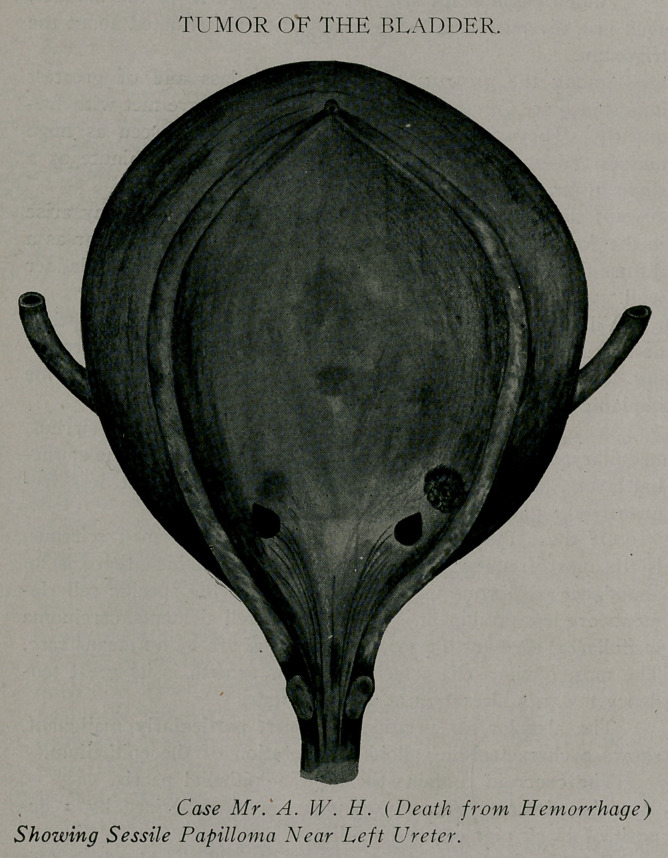


**Figure f2:**